# Proteomic Study of HPV-Positive Head and Neck Cancers: Preliminary Results

**DOI:** 10.1155/2014/430906

**Published:** 2014-03-02

**Authors:** Géraldine Descamps, Ruddy Wattiez, Sven Saussez

**Affiliations:** ^1^Laboratory of Anatomy and Cell Biology, Faculty of Medicine and Pharmacy, University of Mons, Pentagone 2A, Avenue du Champ de Mars 6, 7000 Mons, Belgium; ^2^Laboratory of Proteomics and Microbiology, Faculty of Sciences, University of Mons, Mons, Belgium

## Abstract

Human papillomavirus (HPV) was recently recognized as a new risk factor for head and neck squamous cell carcinoma. For oropharyngeal cancers, an HPV+ status is associated with better prognosis in a subgroup of nonsmokers and nondrinkers. However, HPV infection is also involved in the biology of head and neck carcinoma (HNC) in patients with a history of tobacco use and/or alcohol consumption. Thus, the involvement of HPV infection in HN carcinogenesis remains unclear, and further studies are needed to identify and analyze HPV-specific pathways that are involved in this process. Using a quantitative proteomics-based approach, we compared the protein expression profiles of two HPV+ HNC cell lines and one HPV− HNC cell line. We identified 155 proteins that are differentially expressed (*P* < 0.01) in these three lines. Among the identified proteins, prostate stem cell antigen (PSCA) was upregulated and eukaryotic elongation factor 1 alpha (EEF1**α**) was downregulated in the HPV+ cell lines. Immunofluorescence and western blotting analyses confirmed these results. Moreover, PSCA and EEF1**α** were differentially expressed in two clinical series of 50 HPV+ and 50 HPV− oral cavity carcinomas. Thus, our study reveals for the first time that PSCA and EEF1**α** are associated with the HPV-status, suggesting that these proteins could be involved in HPV-associated carcinogenesis.

## 1. Introduction

Head and neck cancers (HNCs) constitute a heterogeneous group of tumors that often arise in the oral cavity, oropharynx, hypopharynx, and larynx. HNC is the sixth most common cancer, with as many as 466,831 new cases diagnosed in men in 2008 [1]. HNC generally has a poor prognosis; its 5-year survival rate ranges between 40 and 50%. HNC patients usually have histories of heavy tobacco and alcohol consumption. However, the International Agency for Research in Cancer (IARC) has recently recognized human papillomavirus (HPV) as a risk factor for oropharyngeal squamous cell carcinoma (OSCC). Indeed, numerous studies have provided consistent evidence that HPV has an etiologic role in 20 to 50% of OSCCs, and it is associated with a better prognosis in terms of survival and response to therapy [2].

Although the relationship between HPV infection and patient prognosis seems clear in oropharyngeal carcinoma, this relationship is less evident in the other anatomical sites affected by HNC, such as the oral cavity, larynx, and hypopharynx. The meta-analysis performed by Ragin and Taioli, which examined the relationship between HPV and overall survival, did not show any differences between HPV+ and HPV− patients with cancers at nonoropharyngeal sites [3]. Recently, we demonstrated that HPV+ oral SCC patients with a history of tobacco use and/or alcohol consumption have a significantly poorer prognosis compared to HPV− patients [4], and two Swedish studies reported that oral HPV infection is associated with a dramatically increased risk of recurrence in oral SCCs [5, 6]. However, other studies have failed to demonstrate an association between HPV status and prognosis [7–9]. Therefore, it seems clear that the biology of oropharyngeal tumors in younger patients, nondrinkers, and nonsmokers is distinct from that of nonoropharyngeal SCC in older patients and those with a history of tobacco use and/or alcohol consumption [10]. While it is unclear whether tobacco is a risk factor for HPV-induced oropharyngeal tumors, smoking has a negative impact on the survival of HPV+ patients [11]. Thus, researchers agree that there are several possible physiological states according to the patient's HPV infection status, which may or may not be associated with the classical risk factors. Therefore, it is important to understand these differences and the signaling pathways responsible for HPV infection.

Proteomic analysis represents a promising approach for identifying HPV-related signaling pathways. However, a paucity of literature exists regarding the biology of HPV-mediated head and neck tumors. A small number of proteomic studies have been conducted, and these investigations have identified HPV-specific protein candidates in HNC. Additional proteins with altered expression levels were previously identified using 2D electrophoresis followed by mass spectrometry. S100A8, a calcium-binding protein, is a powerful biomarker of HPV18 infection in oral SCC patients [12] and is involved in tumor development and progression [13]. In another study, Melle et al. detected two interesting protein markers that were significantly upregulated in HPV+ oral SCC, TRX and E-FABP [14].

Here, we used a quantitative proteomic-based approach to visualize major changes in protein expression between HPV+ and HPV− HNSCC cell lines. Among these proteins, we selected two candidates to validate our proteomic approach and studied their involvement in the carcinogenesis of HPV+ head and neck cancers. To this end, we performed immunohistochemistry on two clinical series (50 HPV+ oral SCC patients and 50 HPV− oral SCC patients) to support our results. In summary, this study aimed to establish a proteomic signature of HPV infection in head and neck cancer in order to better understand the mechanisms by which HPV drives head and neck carcinogenesis.

## 2. Materials and Methods

### 2.1. Cell Lines

The cell lines used in this study, which were derived from head and neck squamous cell carcinomas, are described in Table 1. Previous to the experiences described below, we performed PCR using E6 and E7 primers to confirm the HPV status of each cell line. The 93VU-174T cell line was obtained from Dr. de Winter (University Medical Center of Amsterdam). The UPCI-SCC-131, Detroit 562, UPCI-SCC-90, and UPCI-SCC-154 cell lines were grown in Minimum Essential Medium (MEM, Gibco Life Technologies, Paisley, UK) supplemented with 10% fetal bovine serum (FBS, Lonza, Verviers, Belgium), 2% L-glutamine (PAA Laboratories, Pasching, Austria), 1% penicillin/streptomycin (PAA Laboratories, Pasching, Austria), and 1% nonessential amino acids (Gibco Life Technologies, Paisley, UK) at 37°C in a humidified 95% air-5% CO_2_ atmosphere. The FaDU and 93VU-147T cell lines were grown in Dulbecco's Modified Eagle Medium (DMEM, Lonza, Verviers, Belgium) supplemented with 10% FBS, 2% L-glutamine, and 1% penicillin/streptomycin at 37°C in a humidified 95% air-5% CO_2_ atmosphere. The culture medium was changed three times each week, and the cells were passaged when they reached 90% confluence. Table 1 presents the characteristics of the cell lines used in this study.

### 2.2. Protein Extraction and Sample Preparation

For total protein extraction, cells were washed twice in cold PBS and centrifuged, and the cell pellets were stored at −80°C. Protein extraction was performed using 6 M guanidinium chloride (lysis buffer from the ICPL kit, SERVA, Germany). The solution was then ultrasonicated for 3 × 10 sec (60% amplitude, U50 IKAtechnik, IMLAB, Boutersem, Belgium) and incubated for 20 min at room temperature. The supernatant was recovered by centrifugation (13,000 rpm for 30 min at 4°C), and the protein concentration was determined according to the Bradford method, using bovine gamma-globulin as a standard.

The proteins were reduced, and their cysteines were alkylated using an ICPL kit (SERVA). The proteins were recovered via acetone precipitation and digested into peptides using trypsin at an enzyme/substrate ratio of 1 : 50 overnight at 37°C. The next day, trypsin digestion was stopped by adding 0.1% formic acid.

### 2.3. Proteomic Analysis: LC MS/MS Analysis

Protein identification and quantification were performed using a label-free strategy on an UHPLC-HRMS platform (Eksigent 2D Ultra and AB SCIEX TripleTOF 5600). The peptides (2 *μ*g) were separated on a 25 cm C18 column (Acclaim PepMap100, 3 *μ*m, Dionex) using a linear gradient (5–35% over 120 min) of acetonitrile (ACN) in water containing 0.1% formic acid at a flow rate of 300 nL min^−1^. To obtain the highest possible retention time stability, which is required for label-free quantification, the column was equilibrated with a 10× volume of 5% ACN before each injection. Mass spectra (MS) were acquired across 400–1500 m/z in high-resolution mode with a 500 msec accumulation time. The precursor selection parameters were as follows: intensity threshold 200 cps, 50 precursors maximum per cycle, 50 msec accumulation time, and 15 sec exclusion after one spectrum. These parameters led to a duty cycle of 3 sec per cycle, ensuring that high-quality extracted ion chromatograms (XICs) were obtained for peptide quantification.

### 2.4. Data Processing

ProteinPilot Software (v4.1) was used to conduct a database search against the UniProt Trembl database (09/30/2011 version), which was restricted to *Homo sapiens* entries. The search parameters included differential amino acid mass shifts for carbamidomethyl cysteine, all biological modifications, amino acid substitutions, and missed trypsin cleavage.

For peptide quantification, PeakView was used to construct XICs for the top 5 peptides of each protein identified with an FDR lower than 1%. Only unmodified and unshared peptides were used for quantification. Peptides were also excluded if their identification confidence was below 0.99, as determined by ProteinPilot. A retention time window of 2 min and a mass tolerance of 0.015 m/z were used. The calculated XICs were exported into MarkerView, and they were normalized based on the summed area of the entire run. Only proteins presenting a fold change higher/lower than 1.5/0.6 with a *P* value lower than 0.05 across the 3 biological replicates analyzed were taken into account for metabolic characterization. Fold changes were assessed using Student's *t*-test. Finally, proteins identified with 1 peptide were validated manually.

### 2.5. Immunofluorescence Staining

Cells were seeded at a density of 5 × 10^5^ cells/well in 12-well plates containing sterile round glass coverslips and grown at 37°C and 5% CO_2_ for 5 days. The cells were washed with PBS and fixed with 4% paraformaldehyde for 15 min. The fixed cells were rinsed with PBS, permeabilized with 0.1% Triton X-100 in PBS for 15 min and blocked with 0.05% casein for 20 min. Then, the cells were treated overnight with primary antibodies against PSCA (Pierce anti-PSCA rabbit polyclonal antibody, Thermo Scientific, Rockford, USA) and EEF1*α* (anti-EEF1A1 rabbit antibody (N-term), Abgent, Huissen, The Netherlands), which were diluted 1 : 50 in blocking solution. The next day, the cells were washed with PBS containing 0.1% Triton X-100 and incubated with Alexa Fluor 488-conjugated anti-rabbit IgG (Invitrogen, Gent, Belgium) for 1 h. The cells were washed with PBS containing 0.1% Triton X-100 for 15 min, rinsed with distilled water for 10 min and mounted with Vectashield Mounting Medium containing DAPI (Vector Laboratories). The cells were observed by confocal microscopy using an Olympus FV1000D laser scanning inverted microscope (Olympus, Hamburg, Germany). The exposure time of each photo was 27.59 s/frame, pictures were captured at 1600 pix/1600 pix, and the pixel time was 10.0 *μ*s/pix. The background noise was adjusted in the same manner and to the same level for each picture. Each picture was analyzed semiquantitatively.

### 2.6. Western Blot Analysis

Proteins were extracted from cells using BugBuster Protein extraction reagent (Novagen, Darmstadt, Germany), and the protein concentrations of the extracts were determined using a Bio-Rad protein assay (BioRad Laboratories, München, Germany). Four microliters 4× LDS sample buffer (NuPAGE, Invitrogen) and 1 *μ*L 20× reducing agent (Fermentas) were added to each protein extract, and the sample volume was brought to 20 *μ*L with deionized water. The samples were heated at 95°C for 5 min, and 30 *μ*g of proteins was separated on 4–20% Mini Protean Gels (BioRad Laboratories, München, Germany). After electrophoresis, the proteins were electrotransferred onto nitrocellulose membranes (Hybond ECL, Amersham). Nonspecific binding sites were blocked by incubation with PBS containing 5% nonfat milk at room temperature for 1 h. Immunodetection was performed overnight at 4°C using anti-EEF1*α* (anti-EEF1A1 rabbit antibody (N-term), Abgent, Huissen, The Netherlands) and anti-PSCA (Pierce anti-PSCA rabbit polyclonal antibody, Thermo Scientific, Rockford, USA) antibodies, which were diluted 1 : 100 in PBS containing 2% nonfat milk. The membrane was washed three times with PBS and incubated for 1 h at room temperature with HRP-conjugated goat anti-rabbit IgG (GE Healthcare Life Sciences, Buckinghamshire, UK), which was diluted in PBS containing 2% nonfat milk. The bound peroxidase was detected using the SuperSignal West Femto kit (Roche), and the bands were visualized by exposing the membranes to photosensitive film (Hyperfilm ECL, Amersham Pharmacia Biotech).

### 2.7. Patients and Tissue Samples

We examined 100 formalin-fixed, paraffin-embedded oral SCC specimens obtained from patients who underwent radical curative surgery between January 2004 and December 2008 at Saint Pieter's Hospital (Brussels) or the EpiCURA Center (Baudour). The tumors were classified according to the TNM classification of the International Union Against Cancer.  Table 2 presents the clinical data of our patients. Among these 100 cases, 50 were HPV+ and 50 were HPV−. This study was approved by the Saint Pieter's Hospital Institutional Review Board (AK/09-09-47/3805AD).

### 2.8. HPV Detection and Typing

HPV detection and typing of paraffin-embedded tissues were performed as described in our previous work [9]. DNA extraction was performed using a QIAamp DNA Mini Kit (Qiagen, Benelux, Belgium), according to the manufacturer's protocol. HPV was detected using PCR with GP5+/GP6+ primers. All DNA extracts were analyzed for the presence of 18 different HPV genotypes using a TaqMan-based real-time quantitative PCR targeting type-specific sequences of the following viral genes: 6 E6, 11 E6, 16 E7, 18 E7, 31 E6, 33 E6, 35 E6, 39 E7, 45 E7, 51 E6, 52 E7, 53 E6, 56 E7, 58 E6, 59 E7, 66 E6, 67 L1, and 68 E7. In each PCR assay, *β*-globin levels were assessed using real-time quantitative PCR to verify the quality of the DNA in the samples and measure the amount of input DNA.

### 2.9. Immunohistochemistry of HPV+ and HPV− Oral Carcinoma Samples

All tumors samples were fixed for 24 h in 10% buffered formaldehyde, dehydrated, and embedded in paraffin. Immunohistochemistry was performed on 5 *μ*m thick sections mounted on silane-coated glass sides. The paraffin-embedded tissue specimens were deparaffinized in toluene, soaked in ethanol, and then soaked in PBS. They were pretreated in a pressure cooker (11 min for PSCA and 6 min for EEF1*α*) in a 10% citrate buffer solution (for EEF1*α*) or a 10% EDTA solution (for PSCA) to unmask the antigens. Then, the sections were incubated in 0.06% hydrogen peroxide for 5 min to block endogenous peroxidase activity, rinsed in PBS, blocked with Protein Block (Serum-Free, Dako, Carpinteria, USA), and incubated at 4°C overnight with rabbit anti-PSCA (Thermo Scientific, Rockford, USA) or anti-EEF1*α* (Bio-Connect, TE Huissen, The Netherlands). The next day, the tissues were incubated with Post Blocking Antibody for 15 min, followed by PowerVision (ImmunoLogic, Duiven, The Netherlands) for 30 min. The slides were washed with PBS between incubation steps. Finally, the localization of the antibody/antigen complex was visualized by staining with DAB (BioGenex, Fremont, USA), and the sections were counterstained with Luxol Fast Blue and mounted with a synthetic medium. To exclude antigen-independent staining, controls, for which the incubation step with the primary antibody was omitted, were examined. In all cases, these controls were negative.

### 2.10. Semiquantitative Immunohistochemical Analysis

Two independent investigators, who were blinded to the clinical details of the patients, assessed PSCA and EEF1*α* immunoreactivities in all tumor areas using an optical microscope (Axiocam MRc5, Zeiss). The mean intensity (MI) was defined as follows: 0 (negative), 1 (weak), 2 (moderate), and 3 (strong). The percentage of immunopositive cells (labeling index, LI) was categorized as follows: 0 (0% positive cells), 1 (1–25%), 2 (26–75%), and 3 (76–100%). Statistical analysis was performed using the Mann-Whitney test to compare the MI and LI values between the HPV+ and HPV− samples.

## 3. Results

### 3.1. Protein Profiling of HPV+ versus HPV− Head and Neck Cancer Cell Lines

Protein profiling using label-free quantification was conducted to identify proteins whose expression was altered by HPV infection. To elucidate the specific effects of HPV in head and neck carcinogenesis and identify potential candidates, we compared the differential patterns of protein expression between one HPV− cell line (FaDU) and two HPV+ cell lines (93VU-147T and UPCI-SCC90). Proteins extracts were analyzed in triplicate for each cell line using tandem mass spectrometry.

For this analysis, we were interested in proteins that had increased or decreased expression levels and are clinically relevant.

Analysis of the three cancer cell lines identified 2221 proteins, among which 155 were differentially expressed between the HPV− and HPV+ cells with significant *P* values of <0.01; 56 of these were downregulated, and 99 were upregulated (Table 3). Two interesting candidates caught our attention due to their known properties and their large fold changes. The expression of prostate stem cell antigen (PSCA) (Accession number: D3DWI6) was reduced 140-fold in the FaDu cells compared to the HPV+ cell lines. Moreover, PSCA has been reported to be oncogenic in some epithelial cells and a tumor suppressor in others. Eukaryotic elongation factor 1 *α* (EEF1*α*) (Accession number: Q6IPS9) expression was four fold higher in the HPV− cells than the HPV+ cell lines. Its upregulation was recently reported to be associated with increased cell proliferation and oncogenic transformation.

### 3.2. PSCA and EEF1*α* Expression in Different HPV+ and HPV− Head and Neck Cancer Cell Lines

**Figure 1:**
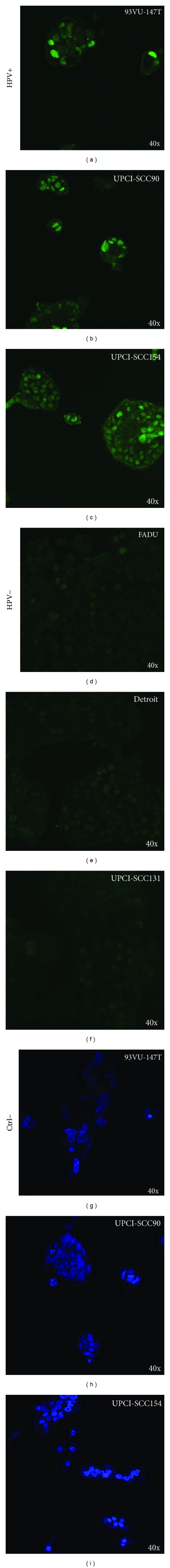
Immunofluorescence staining of PSCA in three HPV+ cell lines ((a), (b), and (c)) and three HPV− cell lines with control DAPI staining ((d), (e), and (f)). Alexa Fluor 488 labeling; confocal microscopy; exposure time of 27.59 s/frame; capture condition of 1600 pix/1600 pix and pixel time of 10.0 *μ*s/pix.

To confirm our mass spectrometry results, we studied the expression of PSCA and EEF1*α* by immunocytochemistry in six head and neck cancer cell lines: 3 HPV+ cell lines (93VU-147T, UPCI-SCC90 and UPCI-SCC154) and 3 HPV− cell lines (FaDU, Detroit and UPCI-SCC131). The results of the immunofluorescence analysis of PSCA in all cell lines are presented in Figure 1. PSCA was mainly nuclear, but it was also distributed at a low level throughout the cytoplasm. PSCA was overexpressed in HPV+ cell lines compared to HPV− cell lines (Figures 1(a), 1(b), and 1(c)). These results are consistent with those obtained in our proteomic analysis.

**Figure 2:**
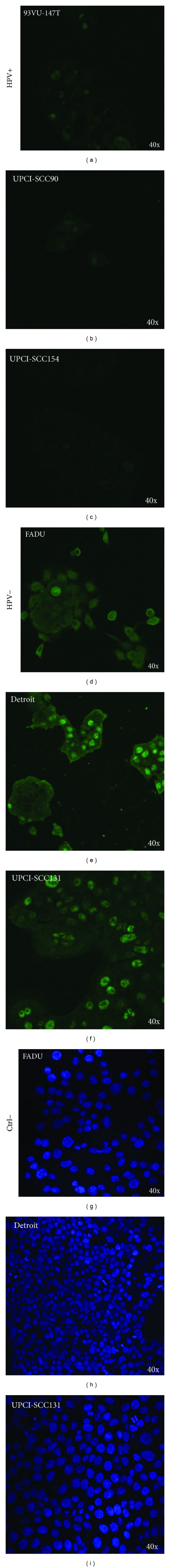
Immunofluorescence staining of EEF1*α* in three HPV+ cell lines with control DAPI staining ((a), (b), and (c)) and three HPV− cell lines ((d), (e), and (f)). Alexa Fluor 488 labeling; confocal microscopy; exposure time of 27.59 s/frame; capture condition of 1600 pix/1600 pix and pixel time of 10.0 *μ*s/pix.

**Figure 3:**
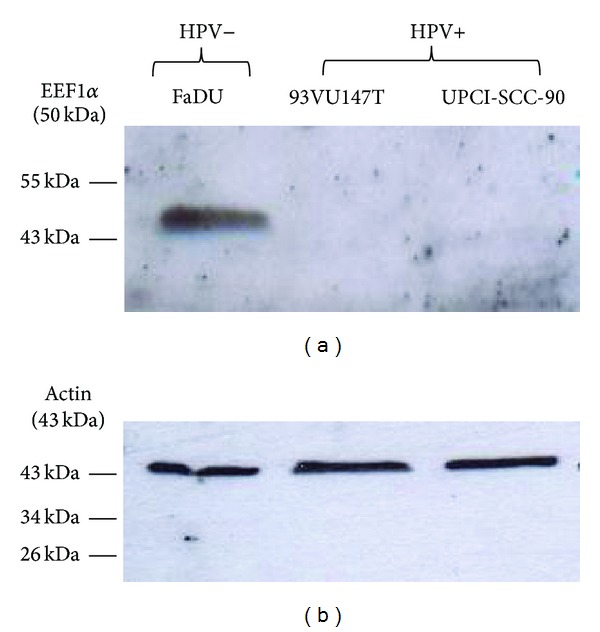
Western blot analysis demonstrating the upregulation of EEF1*α* in the HPV− cell line, FaDu.

Figure 2 illustrates the differential expression of EEF1*α* between HPV+ and HPV− cell lines. EEF1*α* was primarily nuclear, but it was also diffuse throughout the cytoplasm. We also noted a marked difference in the expression of this protein in both cell populations (HPV+ and HPV−). In fact, as expected, confocal microscopy examination of EEF1*α* revealed an increase in the intensity of the immunofluorescence signal in the HPV− cells (Figures 2(d), 2(e), and 2(f)) compared to the HPV+ cells, which showed weak expression of EEF1*α* (Figures 2(a), 2(b), and 2(c)). This observation was validated using western blotting to compare the EEF1*α* expression levels of the cell lines used in our proteomic analysis. In FaDU cell extracts, a band was detected at 50 kDa, which corresponds to the mass of the EEF1*α* protein (Figure 3). This band was not observed in the HPV+ cell lines. We used actin as a loading control, which was detected at 43 kDa in the extracts from all three cell lines (Figure 3). After several attempts, we were not able to validate PSCA expression by western blotting because the primary antibody was not suitable for this technique.

### 3.3. PSCA Protein Expression in Surgical Specimens of OSCC

**Figure 4:**
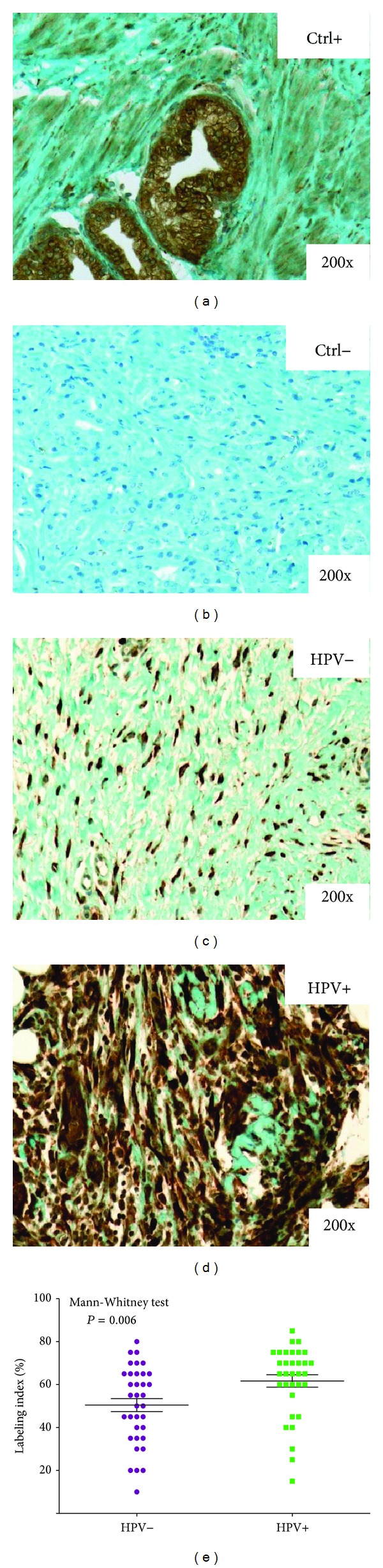
Typical immunohistochemical staining profile of PSCA in HPV− (c) and HPV+ (d) oral tumors. The graph represents the results of the Mann-Whitney test of the PSCA mean labeling index values in the 50 HPV+ and 50 HPV− tumors (e). Panels (a) and (b) show positive and negative controls for PSCA in oral tumors, respectively.

Among the 50 HPV+ cases, qRT-PCR targeting 18 HPV subtypes revealed that 100% of the cases were infected by HPV-16, with two coinfections, HPV-53 and HPV-39. After confirming our results *in vitro*, we evaluated PSCA expression in clinical series of oral cancer. Fifty HPV+ and fifty HPV− oral cancer specimens were examined by immunohistochemistry. As shown in Figure 4(d), PSCA immunostaining was strong in both the cytoplasm and nucleus (Figure 4(d)). To determine whether there was differential protein expression, we compared the two groups (HPV+ versus HPV−) using a nonparametric Mann-Whitney test (Figure 4(e)). PSCA was significantly upregulated in the HPV+ oral tumors compared to the HPV− oral tumors (*P* = 0.006) in terms of the labeling index (LI), which corresponds to the percentage of immunopositive cells.

### 3.4. EEF1*α* Protein Expression in Surgical Specimens of OSCC

**Figure 5:**
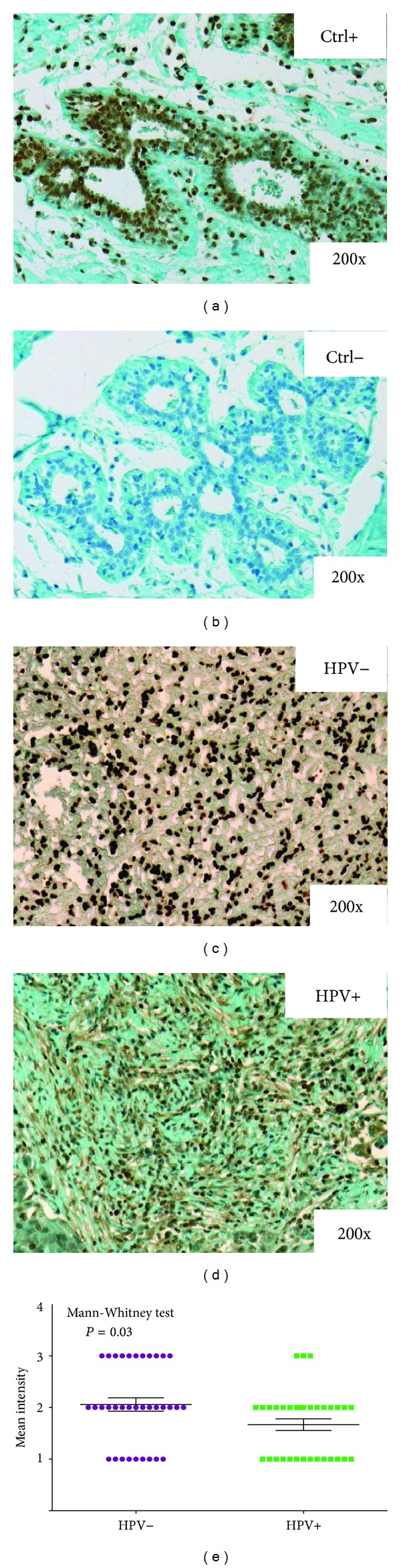
Typical immunohistochemical staining profile of EEF1*α* in HPV− (c) and HPV+ (d) oral tumors. The graph represents the results of the Mann-Whitney test of the EEF1*α* mean labeling index values in the 50 HPV+ and 50 HPV− tumors (e). (a) and (b) show positive and negative controls for EEF1*α* in oral tumors, respectively.

Figures 5(c) and 5(d) present the results of our immunohistochemical analysis of EEF1*α* expression in the same clinical series (50 HPV+ OSCCs *versus* 50 HPV− OSCCs). EEF1*α* was localized in both the nucleus and cytoplasm, but significantly stronger staining intensity was observed in the nucleus (Figure 5(d)). As expected, semiquantitative analysis demonstrated that EEF1*α* expression was increased in HPV− carcinomas compared to HPV+ carcinomas. Indeed, a statistically significant difference in terms of the mean intensity (MI) values between the HPV+ and HPV− tumors was calculated using a nonparametric Mann-Whitney test (*P* = 0.03) (Figure 5(e)).

## 4. Discussion

Recent advances have been made in our understanding of the relationship between head and neck carcinogenesis and HPV. Strong evidence indicates that HPV+ HNSCC comprise a subclass of tumors with a different biology and different clinical properties and that affects specific demographic populations. HPV+ tumors occur in a younger age group, originate more frequently in the oropharynx, and have a lower T stage compared to HPV− tumors [15]. At the histopathological level, we distinguished distinct features of HPV+ tumors, including their identification as nonkeratinizing basal cells and their prominent “koilocytic” morphology [16]. Concerning overall survival, the majority of studies agree that HPV-infected patients have a better prognosis. HPV+ and HPV− tumors also exhibit differences in tumor biology, with HPV+ tumors having fewer p53 mutations and displaying reduced association with tobacco and alcohol consumption [17, 18]. These observations suggest that HPV+ HNSCC and HPV− HNSCC should be considered two distinct cancers with distinct biological pathways: one driven by environmental agents (tobacco and alcohol) and the other driven by infectious agents (high-risk HPV subtypes). However, these two pathologic agents may interact and act synergistically to promote the development of HNSCC.

Despite the progress made in the field of HPV-related HNSCC, a paucity of literature exists with respect to studies investigating the biology of HPV infection in head and neck carcinogenesis. Disease predictors are important from both the clinical and molecular perspectives. Current HNSCC treatments are frequently associated with adverse side effects, and 50% of HNSCC patients die within two years of their initial diagnosis because two-thirds of patients have advanced cancer (stage III or IV) at diagnosis [19, 20]. Therefore, novel approaches are needed to aid clinicians by providing them relevant predictive candidates for the disease to improve patient management. Beyond the clinical challenges, understanding the molecular mechanisms underlying this disease is crucial for developing targeted therapies and individualizing treatment based on the biology of the tumor. In this context, we investigated the global protein expression of three head and neck cancer cell lines, two HPV+ and one HPV−. First, we compared the two populations to identify differences in their proteomic patterns and, consequently, potential candidates of HPV infection. Second, we validated the selected proteins using a clinical series of 100 oral SCC samples (50 HPV+ and 50 HPV−).

Over the past decade, technological advances have been made in the field of proteomics, leading to the identification of specific proteins that are differentially expressed in tumor and control specimens. Mass spectrometry is undoubtedly the most powerful technology for proteomics. The most current mass spectrometers present high resolving power and mass accuracy, allowing for the detection and quantification of thousands of proteins. Thus, clinical proteomics is a powerful diagnostic and prognostic technology. However, advances in the proteomics field have resulted in publications describing numerous potential cancer markers that must be clinically validated prior to the development of a diagnostic test.

In our study, we used liquid chromatography coupled to electrospray ionization tandem mass spectrometry to analyze tryptic peptides from three cell lines (2 HPV+ and 1 HPV−). This technology allowed us to identify and quantify 2221 proteins, among which 155 were differentially expressed between the HPV− and HPV+ cells with significant *P* values of <0.01. The strength of our study lies in the clinical validation of our potential candidates. Indeed, there is a limitation in using cultured cells rather than clinical specimens, as the proteomes of cells grown *in vitro* may not accurately reflect those of *in vivo* cancer cells. However, if the selected protein candidates are further investigated by immunohistochemistry (IHC) using patient tissue samples, the proteomic analysis of cultured cells is entirely valid for the identification of putative candidates. Ye et al. identified 40 differentially expressed proteins between three paired oral SCC cell lines with different metastatic potentials. They were able to confirm their results by IHC and, consequently, identified superoxide dismutase 2 (SOD2) as a predictive marker for the diagnosis of metastasis [21].

Similarly, we validated several of the differentially expressed proteins between the HPV− and HPV+ populations in our study using three different methods. Immunocytochemistry and western blotting confirmed our mass spectrometry results, and IHC also demonstrated those statistically significant differences in 50 HPV+ and 50 HPV− oral SCC specimens. In fact, HPV+ oral carcinomas overexpressed prostate stem cell antigen (PSCA) compared to HPV− oral carcinomas. PSCA was discovered fifteen years ago. It is a glycosylphosphatidylinositol (GPI)-anchored cell surface protein belonging to the Thy-1/Ly-6 family [22]. PSCA was initially identified in prostate cancer but is also expressed in epithelial cells of various organs, such as the bladder, kidney, skin, esophagus, stomach, placenta, and lung [23–26]. Little is known about its physiological functions and signaling cascade, but recently, it was defined as a “Jekyll and Hyde” molecule due to its expression pattern. PSCA seems to act as an oncogene in some cancers, such as prostate, bladder, renal and ovarian carcinomas, and as a tumor suppressor in others, including esophageal and gastric cancer [27]. To date, only one study reported decreased PSCA expression (100-fold) in HNSCC [25].

PSCA seems to be involved in cell growth regulation and to play some roles in signal transduction. Other members of the Ly-6 superfamily are involved in cell adhesion, cell migration, and the regulation of T lymphocyte regulation [28–30]. PSCA overexpression in prostate cancer is related to c-myc amplification [24]. In addition, siRNA-mediated knockdown of PSCA significantly reduces lung cancer cell growth [26]. The same observation was recently made in human prostate cancer cells [31]. Moreover, PSCA is downregulated in gallbladder, esophagus, and stomach tumors [23, 32], as well as our HPV− HNC cell line (FaDU). Therefore, it would be interesting to further validate and explore the clinical implications of PSCA.

Our second candidate protein, EEF1*α*, was overexpressed in the HPV− cell line. EEF1*α* is a GTP-binding protein that interacts with aminoacyl-tRNA to recruit and deliver it to the A site of the ribosome during the elongation phase of protein translation. In addition to its role in protein translation, EEF1*α* is involved in cell migration, cell morphology, protein synthesis, actin cytoskeleton organization, and the modulation of apoptosis sensitivity [33, 34]. Due to its overexpression in many cancers, such as ovarian, breast, lung, and liver cancer, EEF1*α* has been defined as a putative oncogene [35]. This protein is of particular interest because a previous study reported that its downregulation in prostate cancer cells inhibits cell proliferation, invasion, and migration [36]. In contrast, increased EEF1*α* expression is associated with increased cell proliferation, oncogenic transformation, and delayed cell senescence [37–39]. EEF1 also interacts with Akt to modulate its activity and regulate proliferation, survival, and motility in breast cancer cells [40]. Several authors reported that increased expression of this elongation factor is associated with tumorigenesis by enhancing the translation of genes promoting cell growth [38, 41].

To date, no clinical studies have demonstrated the involvement of PSCA or EEF1*α* in head and neck carcinogenesis caused by viral infection, and their functions remain to be elucidated. This study will aid in our understanding of the mechanisms used by HPV to promote the development of head and neck cancers. In conclusion, PSCA and EEF1 meet several criteria, suggesting that they are involved in the biology of HPV-related HNSCC; however, further studies should be conducted to confirm our observations in a larger clinical series. Moreover, it will be interesting to perform functional experiments to understand the signaling pathways disrupted by HPV infection. By silencing several proteins, we plan to study the impact of gene extinction on cell proliferation, migration, invasion, and apoptosis to better understand the mechanisms used by HPV to drive carcinogenesis.

## Figures and Tables

**Table 1:** Description of the characteristics of the cell lines used.

Cell line name	Anatomical site	TNM stage	Sex	HPV status	Origin
FaDU	Hypopharynx	T*x*N*x*M*x*	Male	HPV-negative	ATCC
UPCI-SCC-131	Oral cavity	T2N2M0	Male	HPV-negative	ATCC
Detroit 562	Pharynx	T*x*N*x*M*x*	Female	HPV-negative	ATCC
UPCI-SCC-90	Oropharynx	T2N1M0	Male	HPV-positive	ATCC
93VU-147T	Oral cavity	T4N2	Male	HPV-positive	University Medical Center of Amsterdam
UPCI-SCC-154	Oral cavity	T4N2	Male	HPV-positive	ATCC

**Table 2:** Clinical data of the 100 oral SCC patients.

Variables	Number of cases
Age (years)	
Range	36–90
Mean	58
Sex	
Male	82
Female	18
Anatomic site	
Cheeks	4
Mouth floor	32
Tongue	36
Gums	8
Mandible	5
Palate	2
Retromolar trigone	2
Lips	2
Other	9
Grade (differentiation)	
Well	30
Moderately	51
Poorly	19
TNM stage	
T1-T2	72
T3-T4	28
N stage	
N0	53
N1	12
N2	33
N3	2
Metastasis	
M0	100
M1	0
Risk factors	
Tobacco (90 cases)	
Smoker	67
Nonsmoker	16
Former smoker	7
Alcohol (90 cases)	
Drinker	58
Nondrinker	9
Former drinker	23
Histology	
Bone infiltration	2
Perineural invasion	10
Positive node	19
Capsular evasion	11
Recurrence	
Local	11
Ganglionic	6
Distant metastases	4

**Table 3:** Proteins with decreased and increased abundance between the HPV− cell line and the HPV+ cell lines.

Accession number	*P* value	Fold change	Protein name	Number of peptide identified (95%)
tr∣D3DWI6∣D3DWI6_HUMAN	0.007	0.007	Prostate stem cell antigen	1
tr∣Q6LES2∣Q6LES2_HUMAN	0.000005	0.045	ANXA4 protein	22
tr∣B3KY42∣B3KY42_HUMAN	0.001	0.062	cDNA FLJ46788 fis, clone TRACH3028855, highly similar to Pseudouridylate synthase 7	1
tr∣B4E2Q6∣B4E2Q6_HUMAN	0.003	0.093	Regulation of nuclear pre-mRNA domain-containing protein 2	2
tr∣F8WE04∣F8WE04_HUMAN	0.00156	0.111	Heat shock protein beta-1	54
tr∣Q59GW6∣Q59GW6_HUMAN	0.00375	0.134	Acetyl-CoA acetyltransferase, cytosolic variant	20
tr∣B7Z992∣B7Z992_HUMAN	0.00369	0.137	cDNA FLJ53698, highly similar to Gelsolin	39
tr∣A8K287∣A8K287_HUMAN	0.00793	0.143	Synaptosomal-associated protein	1
tr∣A8K5J7∣A8K5J7_HUMAN	0.00757	0.150	cDNA FLJ77290, highly similar to Homo sapiens BCL2-associated athanogene 5	1
tr∣B2R4I8∣B2R4I8_HUMAN	0.00098	0.159	cDNA, FLJ92106, highly similar to Homo sapiens adaptor-related protein complex 3, sigma 1 subunit(AP3S1),	2
tr∣E9PPU0∣E9PPU0_HUMAN	0.00505	0.168	Epiplakin	97
tr∣B7Z6B8∣B7Z6B8_HUMAN	0.00178	0.171	2,4-dienoyl-CoA reductase, mitochondrial	10
tr∣Q6NVI1∣Q6NVI1_HUMAN	0.00008	0.197	MARCKS protein	26
tr∣C9JEJ2∣C9JEJ2_HUMAN	0.00782	0.199	Choline-phosphate cytidylyltransferase A	15
tr∣Q9BRV4∣Q9BRV4_HUMAN	0.00187	0.202	Vesicle-associated membrane protein 3 (Cellubrevin)	3
tr∣B2RCZ7∣B2RCZ7_HUMAN	0.00005	0.204	Ethylmalonic encephalopathy 1, isoform CRA_a	18
tr∣B4DL87∣B4DL87_HUMAN	0.00444	0.206	cDNA FLJ52243, highly similar to Heat-shock protein beta-1	70
tr∣B7WPG3∣B7WPG3_HUMAN	0.00444	0.208	Heterogeneous nuclear ribonucleoprotein L-like	1
tr∣Q6IAX6∣Q6IAX6_HUMAN	0.00052	0.213	3′-phosphoadenosine 5′-phosphosulfate synthase 1 OS	1
tr∣E7EMB1∣E7EMB1_HUMAN	0.00413	0.227	Switch-associated protein 70	9
tr∣E9PMV1∣E9PMV1_HUMAN	0.00202	0.238	Plectin	10
tr∣F5GXF7∣F5GXF7_HUMAN	0.00479	0.252	Zinc finger protein 185	16
tr∣E5RJR5∣E5RJR5_HUMAN	0.00022	0.252	S-phase kinase-associated protein 1	4
tr∣E7ESP4∣E7ESP4_HUMAN	0.00135	0.259	Integrin alpha-2	12
tr∣Q96IF9∣Q96IF9_HUMAN	0.00454	0.273	VCP protein	112
tr∣F8W785∣F8W785_HUMAN	0.00221	0.276	Golgi integral membrane protein 4	1
tr∣B7Z5V6∣B7Z5V6_HUMAN	0.00881	0.278	cDNA FLJ57046, highly similar to Lysosomal alpha-glucosidase	4
tr∣A6NEL0∣A6NEL0_HUMAN	0.00582	0.279	Non-histone chromosomal protein HMG-14	19
tr∣G0TQY6∣G0TQY6_HUMAN	0.00471	0.281	Lutheran blood group	19
tr∣A8K4W6∣A8K4W6_HUMAN	0.00045	0.287	Phosphoglycerate kinase	123
tr∣Q53RU4∣Q53RU4_HUMAN	0.00154	0.292	Putative uncharacterized protein MSH2	4
tr∣A8K2Y9∣A8K2Y9_HUMAN	0.0011	0.308	6-phosphogluconate dehydrogenase, decarboxylating	33
tr∣B3KMV5∣B3KMV5_HUMAN	0.00078	0.319	cDNA FLJ12728 fis, clone NT2RP2000040, highly similar to Protein FAM62A	9
tr∣B3KMN7∣B3KMN7_HUMAN	0.00131	0.320	cDNA FLJ11717 fis, clone HEMBA1005241	5
tr∣B4DN60∣B4DN60_HUMAN	0.00015	0.323	Asparagine-tRNA ligase, cytoplasmic	10
tr∣B1ANK7∣B1ANK7_HUMAN	0.00465	0.347	Fumarate hydratase	19
tr∣Q0VDC6∣Q0VDC6_HUMAN	0.00257	0.357	FKBP1A protein	9
tr∣C8KIL8∣C8KIL8_HUMAN	0.00769	0.368	Glutathione reductase delta8 alternative splicing variant	1
tr∣B4DUK1∣B4DUK1_HUMAN	0.00161	0.375	cDNA FLJ51310, moderately similar to Peroxiredoxin-6	10
tr∣E9PP14∣E9PP14_HUMAN	0.00564	0.383	GDP-L-fucose synthase	1
tr∣D6RE99∣D6RE99_HUMAN	0.00776	0.399	Histidine triad nucleotide-binding protein 1	8
tr∣B1AKP7∣B1AKP7_HUMAN	0.00099	0.403	TAR DNA binding protein	11
tr∣Q6FHQ6∣Q6FHQ6_HUMAN	0.00442	0.418	IDH1 protein	21
tr∣A8K4I2∣A8K4I2_HUMAN	0.00771	0.421	Histone 1, H1c	123
tr∣A0PK02∣A0PK02_HUMAN	0.00526	0.424	PLXNB2 protein	3
tr∣Q6IAW5∣Q6IAW5_HUMAN	0.00939	0.446	CALU protein	22
tr∣Q6ZNW0∣Q6ZNW0_HUMAN	0.0098	0.449	cDNA FLJ27036 fis, clone SLV08019, highly similar to Homo sapiens stomatin (EPB72)-like 2 (STOML2)	10
tr∣A4UCS8∣A4UCS8_HUMAN	0.00267	0.455	Enolase	130
tr∣Q5TZZ9∣Q5TZZ9_HUMAN	0.00184	0.461	ANXA1 protein	96
tr∣E2DRY6∣E2DRY6_HUMAN	0.00118	0.504	Enolase	217
tr∣A4D105∣A4D105_HUMAN	0.00122	0.514	Replication protein A3, 14 kDa	7
tr∣Q5TCI8∣Q5TCI8_HUMAN	0.00168	0.530	Lamin A/C	119
tr∣B2R5W3∣B2R5W3_HUMAN	0.00065	0.566	cDNA, FLJ92658, highly similar to Homo sapiens poly (ADP-ribose) polymerase family, member 1 (PARP1)	51
tr∣B4E0E1∣B4E0E1_HUMAN	0.00065	0.566	cDNA FLJ53442, highly similar to Poly (ADP-ribose) polymerase 1	52
tr∣D6W5C0∣D6W5C0_HUMAN	0.0096	0.608	Spectrin, beta, nonerythrocytic 1, isoform CRA_b	39
tr∣E9KL44∣E9KL44_HUMAN	0.00912	0.646	Epididymis tissue sperm binding protein	39
tr∣B7Z6F8∣B7Z6F8_HUMAN	0.00713	1.244	Clathrin interactor 1	6
tr∣A8K7A4∣A8K7A4_HUMAN	0.00524	1.414	cDNA FLJ76904, highly similar to Homo sapiens methionine adenosyltransferase II, beta (MAT2B)	12
tr∣F8WDI0∣F8WDI0_HUMAN	0.00874	1.505	Ubiquitin-like-conjugating enzyme ATG3	2
tr∣B3KRT1∣B3KRT1_HUMAN	0.00437	1.706	Inositol-3-phosphate synthase 1	11
tr∣Q6PK50∣Q6PK50_HUMAN	0.0069	1.788	HSP90AB1 protein	65
tr∣Q6NVC0∣Q6NVC0_HUMAN	0.00315	1.80	SLC25A5 protein	37
tr∣E5RH41∣E5RH41_HUMAN	0.00169	1.848	Transcription initiation factor IIE subunit beta	1
tr∣B4DYH1∣B4DYH1_HUMAN	0.00023	1.878	Heat shock 105 kDa/110 kDa protein 1, isoform CRA_b	52
tr∣E9PQI8∣E9PQI8_HUMAN	0.00195	1.888	U4/U6.U5 tri-snRNP-associated protein 1	3
tr∣B5BTY7∣B5BTY7_HUMAN	0.00798	1.966	T-complex protein 1 subunit beta	46
tr∣B4DUG4∣B4DUG4_HUMAN	0.00275	1.975	cDNA FLJ51308	1
tr∣B3KTJ9∣B3KTJ9_HUMAN	0.00804	1.975	cDNA FLJ38393 fis, clone FEBRA2007212	15
tr∣D6R938∣D6R938_HUMAN	0.00024	1.986	Calcium/calmodulin-dependent protein kinase (CaM kinase) II delta	2
tr∣A8K259∣A8K259_HUMAN	0.00046	2.070	cDNA FLJ78501, highly similar to Homo sapiens serpin peptidase inhibitor, clade H (heat shock protein 47), member 1, (collagen binding protein 1) (SERPINH1)	18
tr∣Q54A51∣Q54A51_HUMAN	0.00291	2.079	Basigin (Ok blood group), isoform CRA_a	20
tr∣Q6IPH7∣Q6IPH7_HUMAN	0.00287	2.131	RPL14 protein	19
tr∣A8K9U6∣A8K9U6_HUMAN	0.00504	2.132	cDNA FLJ76121, highly similar to Homo sapiens zinc finger CCCH-type, antiviral 1 (ZC3HAV1)	7
tr∣D3DPU2∣D3DPU2_HUMAN	0.00049	2.135	Adenylyl cyclase-associated protein	59
tr∣B3KN49∣B3KN49_HUMAN	0.00667	2.165	cDNA FLJ13562 fis, clone PLACE1008080, highly similar to Homo sapiens hexamethylene bis-acetamide inducible 1 (HEXIM1)	6
tr∣E9PR70∣E9PR70_HUMAN	0.0005	2.167	Serpin H1	17
tr∣Q05D43∣Q05D43_HUMAN	0.00597	2.195	YBX1 protein	25
tr∣E7EQV9∣E7EQV9_HUMAN	0.00711	2.247	Ribosomal protein L15	5
tr∣A8K2Q6∣A8K2Q6_HUMAN	0.00501	2.278	Peptidyl-prolyl cis-trans isomerase	3
tr∣Q05CM9∣Q05CM9_HUMAN	0.000005	2.287	PSIP1 protein	18
tr∣Q7L7Q6∣Q7L7Q6_HUMAN	0.00109	2.321	RTN4	6
tr∣Q5U077∣Q5U077_HUMAN	0.00247	2.379	L-lactate dehydrogenase	38
tr∣F5GZA8∣F5GZA8_HUMAN	0.00582	2.384	SH3 domain-binding protein 1	6
tr∣E7EPK6∣E7EPK6_HUMAN	0.00396	2.394	40S ribosomal protein S24	7
tr∣Q8TBR3∣Q8TBR3_HUMAN	0.00685	2.414	Fusion (Involved in t(12;16) in malignant liposarcoma)	33
tr∣A8MX94∣A8MX94_HUMAN	0.00964	2.475	Glutathione S-transferase P	50
tr∣B4DUI3∣B4DUI3_HUMAN	0.00619	2.484	Eukaryotic translation initiation factor 3 subunit J	10
tr∣Q6P1N4∣Q6P1N4_HUMAN	0.00019	2.492	IQGAP1 protein	64
tr∣A4QPB0∣A4QPB0_HUMAN	0.00019	2.492	IQ motif containing GTPase activating protein 1	83
tr∣B7ZBH1∣B7ZBH1_HUMAN	0.00876	2.499	Eukaryotic translation initiation factor 6	9
tr∣B4DZI8∣B4DZI8_HUMAN	0.00931	2.525	Coatomer protein complex, subunit beta 2	8
tr∣B4DWA0∣B4DWA0_HUMAN	0.00454	2.535	cDNA FLJ54188, moderately similar to High mobility group protein HMG-I/HMG-Y	8
tr∣B4DY28∣B4DY28_HUMAN	0.0063	2.556	cDNA FLJ61189, highly similar to Cysteine and glycine-rich protein 1	2
tr∣B7Z921∣B7Z921_HUMAN	0.00179	2.601	cDNA FLJ61669, highly similar to Transcription elongation regulator 1	5
tr∣Q8NF45∣Q8NF45_HUMAN	0.00368	2.657	FLJ00353 protein	8
tr∣F8WEE0∣F8WEE0_HUMAN	0.00313	2.672	Protein NDRG1	1
tr∣E9PIM9∣E9PIM9_HUMAN	0.00026	2.676	Ribonuclease H1	24
tr∣B4E2D3∣B4E2D3_HUMAN	0.00154	2.685	Nuclear pore complex protein Nup50	4
tr∣Q6FI03∣Q6FI03_HUMAN	0.00472	2.715	G3BP protein	27
tr∣B7Z7L3∣B7Z7L3_HUMAN	0.00077	2.752	NADH-cytochrome b5 reductase 3	10
tr∣B1AH89∣B1AH89_HUMAN	0.00436	2.775	Tubulin tyrosine ligase-like family, member 12	14
tr∣Q6IBT3∣Q6IBT3_HUMAN	0.00347	2.783	CCT7 protein	37
tr∣Q53HV2∣Q53HV2_HUMAN	0.00347	2.783	Chaperonin containing TCP1, subunit 7 (Eta) variant	45
tr∣Q5W0H4∣Q5W0H4_HUMAN	0.00403	2.793	Tumor protein, translationally controlled 1	13
tr∣B4DZX7∣B4DZX7_HUMAN	0.00318	2.817	Thioredoxin domain containing, isoform CRA_b	1
tr∣B3KRA1∣B3KRA1_HUMAN	0.00016	2.844	cDNA FLJ33914 fis, clone CTONG2016575, highly similar to SON PROTEIN	3
tr∣B7Z8R6∣B7Z8R6_HUMAN	0.00049	2.846	cDNA FLJ51445, highly similar to AMBP protein	1
tr∣Q6IAX2∣Q6IAX2_HUMAN	0.0057	2.852	RPL21 protein	13
tr∣B2R4F3∣B2R4F3_HUMAN	0.00842	2.868	cDNA, FLJ92068, highly similar to Homo sapiens Rho GDP dissociation inhibitor (GDI) beta (ARHGDIB)	3
tr∣D3DQ70∣D3DQ70_HUMAN	0.00312	2.882	SERPINE1 mRNA binding protein 1, isoform CRA_d	15
tr∣E7ERF4∣E7ERF4_HUMAN	0.00326	2.973	Adenylosuccinate lyase	10
tr∣B2RAU8∣B2RAU8_HUMAN	0.00573	3.071	cDNA, FLJ95131, highly similar to Homo sapiens nucleolar and coiled-body phosphoprotein 1 (NOLC1)	11
tr∣B4DIT0∣B4DIT0_HUMAN	0.00069	3.098	Anion exchange protein 2	2
tr∣B5MCA4∣B5MCA4_HUMAN	0.00511	3.111	Epithelial cell adhesion molecule	4
tr∣Q6GMS8∣Q6GMS8_HUMAN	0.00075	3.140	Syntaxin-16	2
tr∣B3KN82∣B3KN82_HUMAN	0.00899	3.209	cDNA FLJ13913 fis, clone Y79AA1000231, highly similar to Nucleolar protein NOP5	12
tr∣B4E0L0∣B4E0L0_HUMAN	0.00362	3.211	cDNA FLJ54030, highly similar to Polymerase delta-interacting protein 3	9
tr∣D3DSF7∣D3DSF7_HUMAN	0.00302	3.236	SON DNA binding protein, isoform CRA_b	7
tr∣F8VVL1∣F8VVL1_HUMAN	0.00199	3.278	Density-regulated protein	6
tr∣A8K787∣A8K787_HUMAN	0.0069	3.285	cDNA FLJ75273, highly similar to Homo sapiens solute carrier family 25 (mitochondrial carrier; adenine nucleotide translocator), member 4	17
tr∣B4DSL9∣B4DSL9_HUMAN	0.00403	3.297	cDNA FLJ58748, highly similar to U3 small nucleolar RNA-associated protein 6homolog	2
tr∣B3KWL6∣B3KWL6_HUMAN	0.00036	3.382	Methionine aminopeptidase	7
tr∣B3KPR5∣B3KPR5_HUMAN	0.00165	3.421	cDNA FLJ32094 fis, clone OCBBF2000986, highly similar to Homo sapiens elongation factor Tu GTP binding domain containing 1, transcript variant 1	1
tr∣Q14222∣Q14222_HUMAN	0.00673	3.562	EEF1A protein	108
tr∣Q16577∣Q16577_HUMAN	0.00673	3.562	Elongation factor 1-alpha	143
tr∣Q53H88∣Q53H88_HUMAN	0.0034	3.579	Dynactin 2 variant	7
tr∣Q59GP5∣Q59GP5_HUMAN	0.00198	3.601	Eukaryotic translation elongation factor 1 alpha 2 variant	45
tr∣Q68CS0∣Q68CS0_HUMAN	0.00079	3.630	Ornithine aminotransferase, mitochondrial	7
tr∣F5GXR3∣F5GXR3_HUMAN	0.00387	3.972	Parathymosin	1
tr∣F5H8L6∣F5H8L6_HUMAN	0.00141	3.996	Dipeptidyl peptidase 3	17
tr∣Q6IPS9∣Q6IPS9_HUMAN	0.00459	4.001	Elongation factor 1-alpha	311
tr∣F8W940∣F8W940_HUMAN	0.00537	4.120	CUGBP Elav-like family member 1	3
tr∣B7ZLC9∣B7ZLC9_HUMAN	0.00416	4.234	GEMIN5 protein	3
tr∣Q6FIG4∣Q6FIG4_HUMAN	0.00017	4.372	RAB1B protein	19
tr∣F5H4R6∣F5H4R6_HUMAN	0.00067	4.373	Nucleosome assembly protein 1-like 1	32
tr∣Q6PK82∣Q6PK82_HUMAN	0.00008	4.425	AP3D1 protein	5
tr∣B3KW52∣B3KW52_HUMAN	0.0073	4.443	cDNA FLJ42145 fis, clone TESTI4000228, highly similar to Mus musculus ubiquitin family domain containing 1 (Ubfd1), mRNA	2
tr∣E9PS95∣E9PS95_HUMAN	0.00885	4.636	Mitochondrial glutamate carrier 1	1
tr∣Q6FH57∣Q6FH57_HUMAN	0.00045	4.653	Peptidyl-prolyl cis-trans isomerase	4
tr∣B3KN79∣B3KN79_HUMAN	0.00107	4.681	cDNA FLJ13894 fis, clone THYRO1001671, highly similar to 59 kDa 2′-5′-oligoadenylate synthetase-like protein	3
tr∣Q53GW1∣Q53GW1_HUMAN	0.00487	4.697	Vesicle transport-related protein isoform a variant (Fragment)	3
tr∣Q5U0I6∣Q5U0I6_HUMAN	0.00098	4.810	RAB1A protein	14
tr∣Q5TBU5∣Q5TBU5_HUMAN	0.00439	5.144	Adipose specific 2	1
tr∣E9PK25∣E9PK25_HUMAN	0.000007	6.239	Cofilin-1	96
tr∣F8W7I9∣F8W7I9_HUMAN	0.00003	6.928	Ran GTPase-activating protein 1	15
tr∣B3KU10∣B3KU10_HUMAN	0.00049	7.509	Interferon-induced GTP-binding protein Mx1	22
tr∣Q75MY7∣Q75MY7_HUMAN	0.0005	7.948	MX2	11
tr∣D2KFR9∣D2KFR9_HUMAN	0.00057	8.090	Signal transducer and activator of transcription 1-alpha/beta	3
tr∣E9PCQ3∣E9PCQ3_HUMAN	0.00031	8.511	Ubiquitin carboxyl-terminal hydrolase	1
tr∣B4DTE6∣B4DTE6_HUMAN	0.0066	8.644	cDNA FLJ56243, highly similar to Melanoma-associated antigen 4	6
tr∣Q8IV97∣Q8IV97_HUMAN	0.00356	10.882	Solute carrier family 7 (Cationic amino acid transporter, y+ system), member 5	3
tr∣Q96J85∣Q96J85_HUMAN	0.00055	14.133	C-Mpl binding protein	1
tr∣F5H667∣F5H667_HUMAN	0.00003	16.263	Aspartyl/asparaginyl beta-hydroxylase	4
tr∣A5GZA6∣A5GZA6_HUMAN	0.000002	53.611	Cysteine-rich with EGF-like domain protein 2	2
tr∣B4DZM8∣B4DZM8_HUMAN	0.00002	145.941	26S proteasome non-ATPase regulatory subunit 5	2
